# Supplementary material for: Artificial Intelligence to Facilitate Clinical Trial Recruitment in Age-Related Macular Degeneration
Source: Ophthalmol Sci. 2024 Jun 19;4(6):100566. doi: 10.1016/j.xops.2024.100566 (PMC11321286; doi:10.1016/j.xops.2024.100566)

Supplemental Figure 11. **Examples of our fovea location approximation.** Images on the top row are of the AI neurosensory segmentation map; images on the bottom row are the associated OCT en-face projection for the same scan. Comparison is between (i) assuming the fovea is located at the image center (blue cross) and (ii) the minima of the AI neurosensory retina segmentation map (white plus) with (iii) the clinician reference (gold star). The delta indicates the distance between the AI-determined location and clinician reference.

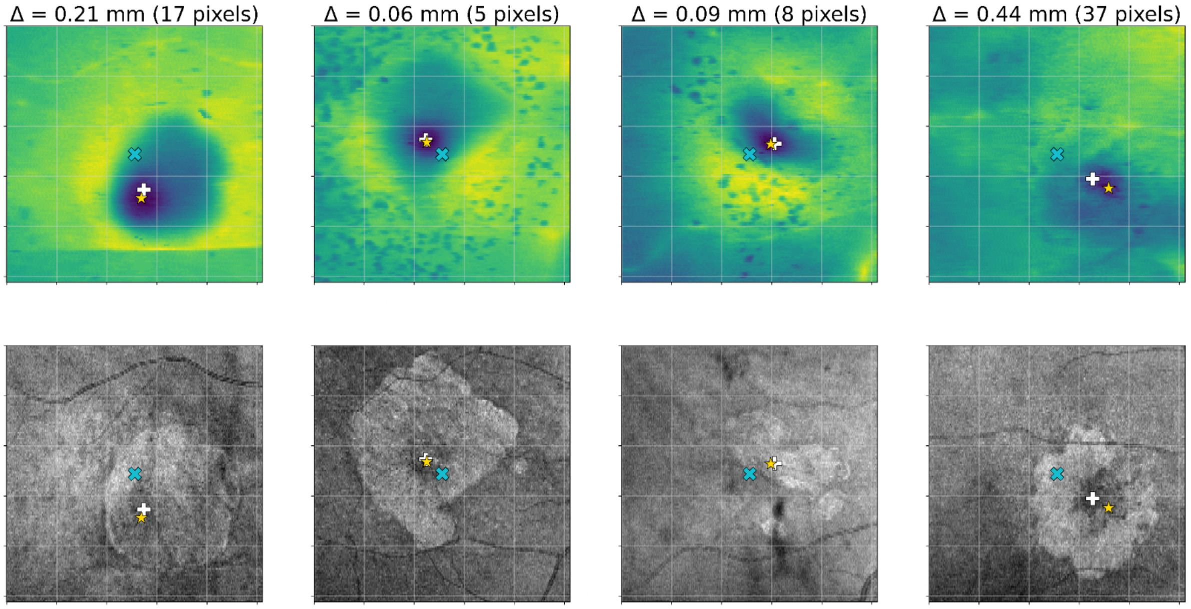

Supplement: Supplemental Figure 11 [file mmc3.pdf]
