# Supplementary material for: Artificial Intelligence to Facilitate Clinical Trial Recruitment in Age-Related Macular Degeneration
Source: Ophthalmol Sci. 2024 Jun 19;4(6):100566. doi: 10.1016/j.xops.2024.100566 (PMC11321286; doi:10.1016/j.xops.2024.100566)

Supplemental Figure 2. **Distribution of ten common retinal conditions as they appear in the dataset.** Each condition was identified via a keyword search in the clinical letters. Percentages were calculated based on the total number of patients passing the inclusion criteria.

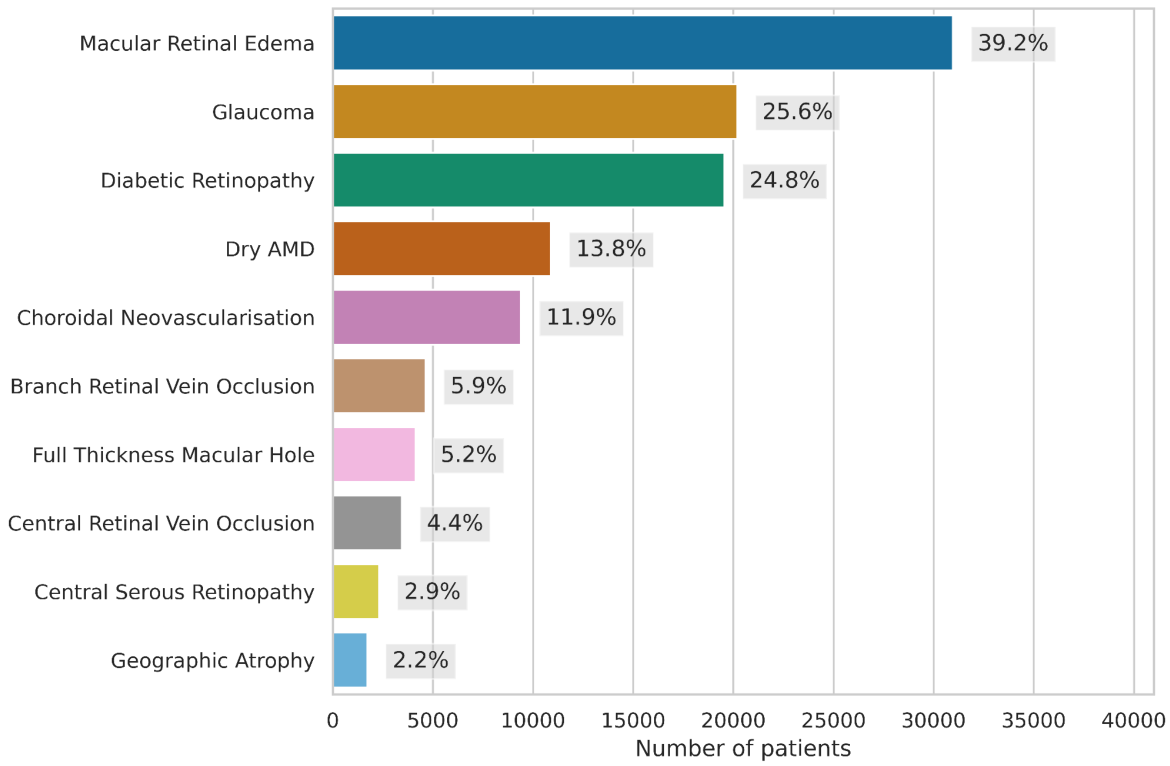

Supplement: Supplemental Figure 2 [file mmc4.pdf]
