# Supplementary material for: Artificial Intelligence to Facilitate Clinical Trial Recruitment in Age-Related Macular Degeneration
Source: Ophthalmol Sci. 2024 Jun 19;4(6):100566. doi: 10.1016/j.xops.2024.100566 (PMC11321286; doi:10.1016/j.xops.2024.100566)

Supplemental Figure 5. Bland-Altman analysis between AI and graders. (a) Bland-Altman plot comparing GA area computed by the AI on the OCT versus the clinician segmented area on the FAF. A_{human} indicates the average measurement of both graders. **(b)** Bland-Altman plot comparing GA area segmented by graders R.C. and M.J. on the FAF. The mean difference and 95% limits of agreement are indicated, calculated based on data points eligible for HORIZON.

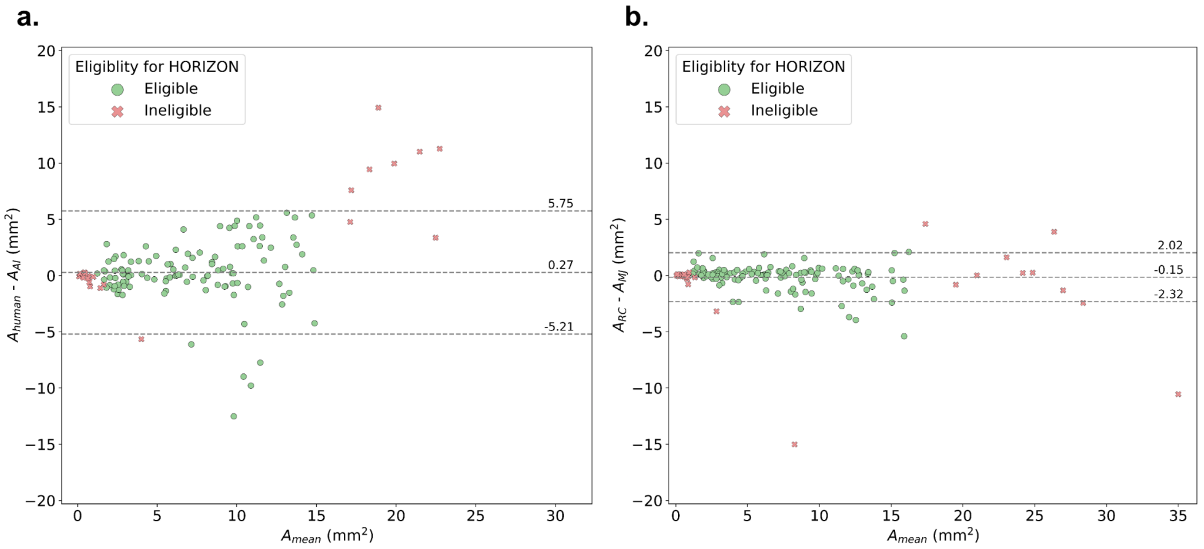

Supplement: Supplemental Figure 5 [file mmc5.pdf]
