# Supplementary material for: Artificial Intelligence to Facilitate Clinical Trial Recruitment in Age-Related Macular Degeneration
Source: Ophthalmol Sci. 2024 Jun 19;4(6):100566. doi: 10.1016/j.xops.2024.100566 (PMC11321286; doi:10.1016/j.xops.2024.100566)

Supplemental Figure 6. Histogram of AI-segmented GA areas. Histogram comparing the AI-segmented GA area for individuals who were determined to (i) have GA and (ii) not have CNV from the classification outputs. The hashed areas indicate the proportion of patients with an affirmative result for ‘geographic atrophy’ in the EHR search.

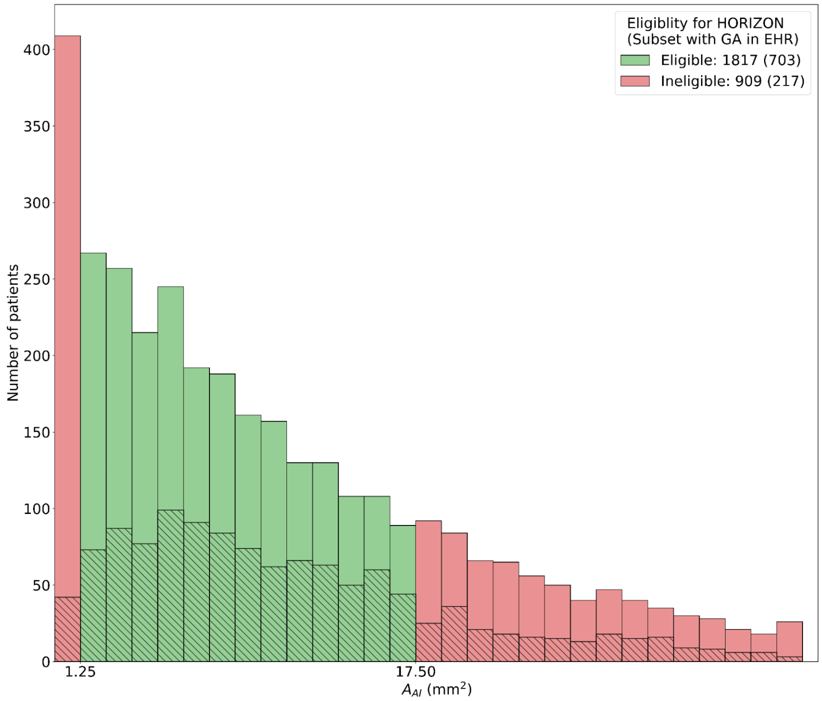

Supplement: Supplemental Figure 6 [file mmc6.pdf]
