# Supplementary material for: Artificial Intelligence to Facilitate Clinical Trial Recruitment in Age-Related Macular Degeneration
Source: Ophthalmol Sci. 2024 Jun 19;4(6):100566. doi: 10.1016/j.xops.2024.100566 (PMC11321286; doi:10.1016/j.xops.2024.100566)

Supplemental Figure 7. Examples of the AI-segmented OCT underestimating the clinician-segmented FAF. Shown are four examples above the upper limit of agreement in **Figure S2** (a). Images on the top row are the OCT en-face overlaid with the AI segmentation; images on the bottom row are the associated FAF image from the same eye overlaid with the segmentation from grader R.C. The AI-determined fovea location (white plus) and a 1500 µm circle centered on the fovea (red circle) are indicated. The AI and averaged human GA area estimations are indicated.

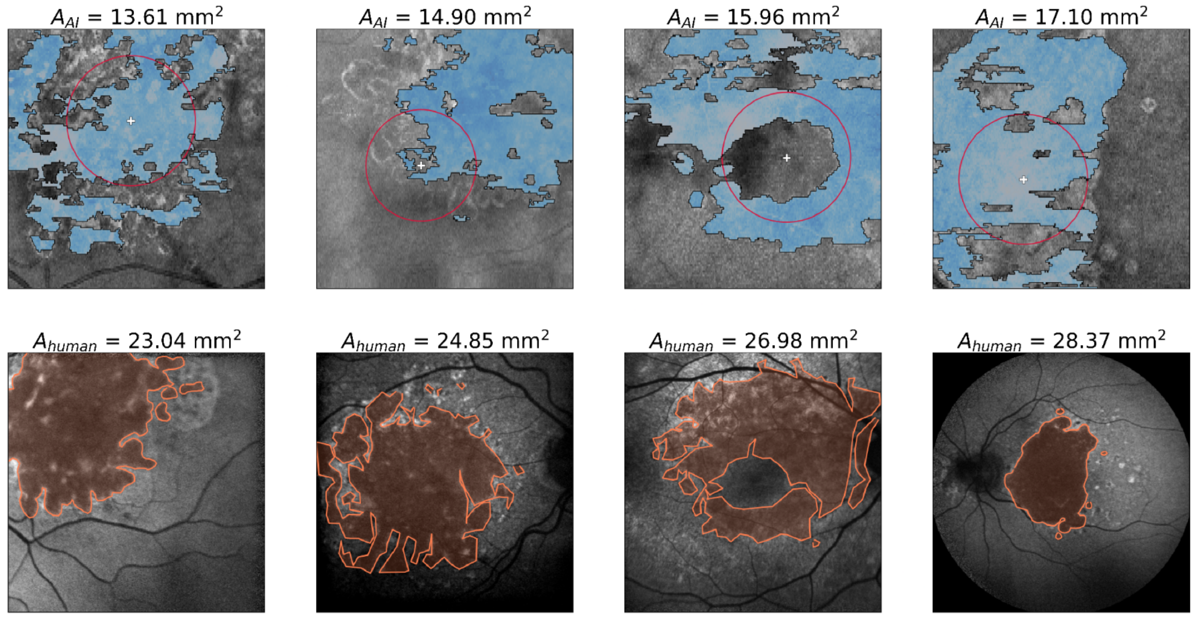

Supplement: Supplemental Figure 7 [file mmc7.pdf]
