# Supplementary material for: Artificial Intelligence to Facilitate Clinical Trial Recruitment in Age-Related Macular Degeneration
Source: Ophthalmol Sci. 2024 Jun 19;4(6):100566. doi: 10.1016/j.xops.2024.100566 (PMC11321286; doi:10.1016/j.xops.2024.100566)

Supplemental Figure 9. **Receiver operating characteristic curves from the AI system.** These were created by comparing AI predictions to the clinician-graded ground truth over the threshold tuning set.

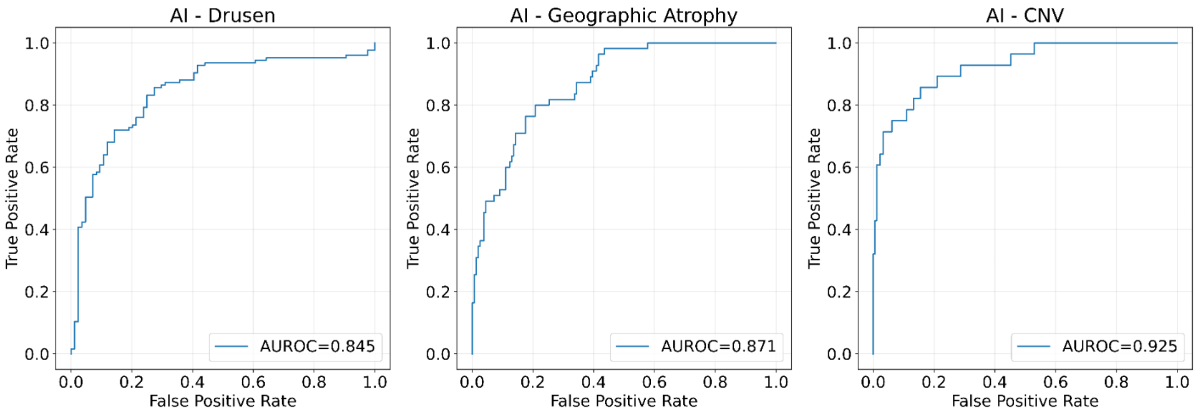

Supplement: Supplemental Figure 9 [file mmc9.pdf]
