# Supplementary material for: Artificial Intelligence to Facilitate Clinical Trial Recruitment in Age-Related Macular Degeneration
Source: Ophthalmol Sci. 2024 Jun 19;4(6):100566. doi: 10.1016/j.xops.2024.100566 (PMC11321286; doi:10.1016/j.xops.2024.100566)
Supplement: Supplemental Table 1 [file mmc10.pdf]

Supplemental Table 1. **Eligibility criteria to shortlist patients for each trial with the AI system.** We define 'central' as being where at least 10% of the pixels in a 1500 μm radius circular area centered on the fovea contain GA. We define 'fovea involving' as being where 100% of the pixels in a 50 μm radius circular area centered on the fovea contain GA. The number of patients shortlisted according to each criterion is also shown.

		**Clinical trials**			
		**HORIZON**[26] (Novartis, Gyroscope)	**DERBY**[27] (Apellis)	**JNJ-81201887**[29] (Janssen)	**GATHER2**[28] (IVERIC bio)
**Eligibility criteria**	**Age**	≥ 55 years	≥ 60 years	≥ 60 years	≥ 50 years
	**CNV**	Not permitted in study eye	Not permitted in study eye	N/A	Not permitted in either eye
	**GA:** secondary to AMD	Required	Required	Required	Required
	**GA:** area (mm^2)	1.25–17.5	2.50–17.5	2.50–17.5	2.50–17.5
	**GA:** central	N/A	N/A	N/A	Required[36]
	**GA:** fovea involving	N/A	N/A	Not permitted	Not permitted
**Number of patients shortlisted by AI**		1,817	1,580	768	438
