# Supplementary material for: Artificial Intelligence to Facilitate Clinical Trial Recruitment in Age-Related Macular Degeneration
Source: Ophthalmol Sci. 2024 Jun 19;4(6):100566. doi: 10.1016/j.xops.2024.100566 (PMC11321286; doi:10.1016/j.xops.2024.100566)
Supplement: Supplemental Table 3 [file mmc11.pdf]

Supplemental Table 3. **Estimated proportions of eligible individuals of those shortlisted.** Inference of the proportion of eligible individuals out of all those shortlisted for each strategy. 95% CIs were extrapolated from the confidence intervals calculated for the clinical validation.

	**Number of patients shortlisted**	**Number of patients eligible** (95% CI)	**% of patients eligible** (95% CI)
**EHR search**	1729	693 (677 - 719)	40 (39 - 42)
**AI**	1817	1139 (978 - 1281)	63 (54 - 71)
**EHR search + AI**	703	604 (555 - 646)	86 (79 - 92)
