# Supplementary material for: Artificial Intelligence to Facilitate Clinical Trial Recruitment in Age-Related Macular Degeneration
Source: Ophthalmol Sci. 2024 Jun 19;4(6):100566. doi: 10.1016/j.xops.2024.100566 (PMC11321286; doi:10.1016/j.xops.2024.100566)
Supplement: Supplemental Table 5 [file mmc12.pdf]

Supplemental Table 5. **Patients filtered into validation strata.** Each cell contains the number of patients from the initial cohort belonging to each validation stratum.

**Number of patients shortlisted**	(1) **EHR contains 'geographic atrophy'**	(2) **EHR does not contain 'geographic atrophy'**
(A) **AI predicts eligible**	703	1114
(B) **AI predicts ineligible**	1026	76471
